# SalK/SalR, a Two-Component Signal Transduction System, Is Essential for Full Virulence of Highly Invasive *Streptococcus suis* Serotype 2

**DOI:** 10.1371/journal.pone.0002080

**Published:** 2008-05-07

**Authors:** Ming Li, Changjun Wang, Youjun Feng, Xiuzhen Pan, Gong Cheng, Jing Wang, Junchao Ge, Feng Zheng, Min Cao, Yaqing Dong, Di Liu, Jufang Wang, Ying Lin, Hongli Du, George F. Gao, Xiaoning Wang, Fuquan Hu, Jiaqi Tang

**Affiliations:** 1 Department of Microbiology, Third Military Medical University, Chongqing, China; 2 Department of Epidemiology, Research Institute for Medicine of Nanjing Command, Nanjing, China; 3 Center for Molecular Immunology, Institute of Microbiology, Chinese Academy of Sciences, Beijing, China; 4 Provincial Key Laboratory of Biotechnology, School of Biosciences & Bioengineering, South China University of Technology, Guangzhou, China; 5 Graduate University, Chinese Academy of Sciences, Beijing, China; Theodor-Boveri-Institut fur Biowissenschaften, Wurzburg, Germany

## Abstract

**Background:**

*Streptococcus suis* serotype 2 (*S. suis* 2, SS2) has evolved into a highly infectious entity, which caused the two recent large-scale outbreaks of human SS2 epidemic in China, and is characterized by a toxic shock-like syndrome. However, the molecular pathogenesis of this new emerging pathogen is still poorly understood.

**Methodology/Principal Findings:**

89K is a newly predicted pathogenicity island (PAI) which is specific to Chinese epidemic strains isolated from these two SS2 outbreaks. Further bioinformatics analysis revealed a unique two-component signal transduction system (TCSTS) located in the candidate 89K PAI, which is orthologous to the SalK/SalR regulatory system of *Streptococcus salivarius*. Knockout of *salKR* eliminated the lethality of SS2 in experimental infection of piglets. Functional complementation of *salKR* into the isogenic mutant Δ*salKR* restored its soaring pathogenicity. Colonization experiments showed that the Δ*salKR* mutant could not colonize any susceptible tissue of piglets when administered alone. Bactericidal assays demonstrated that resistance of the mutant to polymorphonuclear leukocyte (PMN)-mediated killing was greatly decreased. Expression microarray analysis exhibited a transcription profile alteration of 26 various genes down-regulated in the Δ*salKR* mutant.

**Conclusions/Significance:**

These findings suggest that SalK/SalR is requisite for the full virulence of ethnic Chinese isolates of highly pathogenic SS2, thus providing experimental evidence for the validity of this bioinformatically predicted PAI.

## Introduction

*Streptococcus suis* (*S. suis*) is considered an important zoonotic pathogen causing a variety of life-threatening infections that include meningitis, arthritis, septicaemia and even sudden death in pigs and humans [1], [2]. Among the known 35 serotypes [1], [2], *S. suis* serotype 2 (*S. suis* 2 or SS2) is the most virulent and the most frequently isolated serotype. It was previously thought that SS2 caused only sporadic cases of meningitis and sepsis in humans [1], [2]. However, two major emerging infectious disease outbreaks of SS2 in China (one in Jiangsu Province, 1998, and the other in Sichuan Province, 2005), raised considerable international concerns among the public health professionals [3], [4]. A key feature of these two Chinese outbreaks is the prevalence of a toxic shock-like syndrome manifesting itself as acute high fever, multiple organ failures, short course of disease and high lethality [5]. Despite the growing significance of such infections, little is known about the factors that govern the physiological responses of this emerging organism, especially the genetic repertoire that the streptococcus employs to cause the toxic shock-like syndrome.

To shed light on the mystery of high virulence of the epidemic outbreak strains of SS2, our joint research group completed a comprehensive study of comparative genomics, decoding the whole genome sequences of two virulent SS2 strains (98HAH12 and 05ZYH33) isolated from Chinese infected patients [6]. A candidate pathogenicity island (PAI) called 89K was predicted, which is only present in the epidemic strains in these two SS2 outbtreaks but not in other domestic clinical isolates or international virulent strains [6]. However, this bioinformatically predicted candidate PAI needs experimental validation and its linkage to SS2-related high pathogenicity remains unknown. As a subgroup of genomic islands (GIs), PAI usually contains some distinct genetic elements acquired by horizontal gene transfer [7], [8]. A wide range of molecular machineries such as quorum sensing, TCSTS, and ABC transporters, are often involved in a putative PAI, whereby the PAI responds to environmental signals and fulfills its critical functions contributing to the virulence in pathogens[8]–[10]. Further bioinformatics analysis of the 89K island revealed a distinct two-component signal transduction system (TCSTS) encoded therein appears to be orthologous to the SalK/SalR system of *S. salivarius*, a salivaricin regulated TCSTS [11]. TCSTS, composed of a membrane sensor (histidine kinase, HK) and a cytoplasmic response regulator (RR), is an important mechanism used by bacteria to adapt to and survive in the changing environments. In pathogens, various members of TCSTSs have been shown to play critical roles in both metabolism and pathogenesis [12]–[23]. Up until now, only one orphan response regulator, RevS, has been found to be implicated in the pathogenesis of SS2 [24].

To date, SalK/SalR is still a little-reported TCSTS in the fast-growing field of bacterial pathogenesis. In the present study, we attempted to generate the deletion mutant of *salKR* and assess the contribution of this TCSTS to the high pathogenicity of Chinese SS2 strains. Virulence assays together with a series of experiments enabled us to identify a novel genetic determinant which is required for the overall virulence of Chinese isolates of highly pathogenic SS2.

## Results

### Discovery and Characterization of SalK/SalR

**Figure 1:**
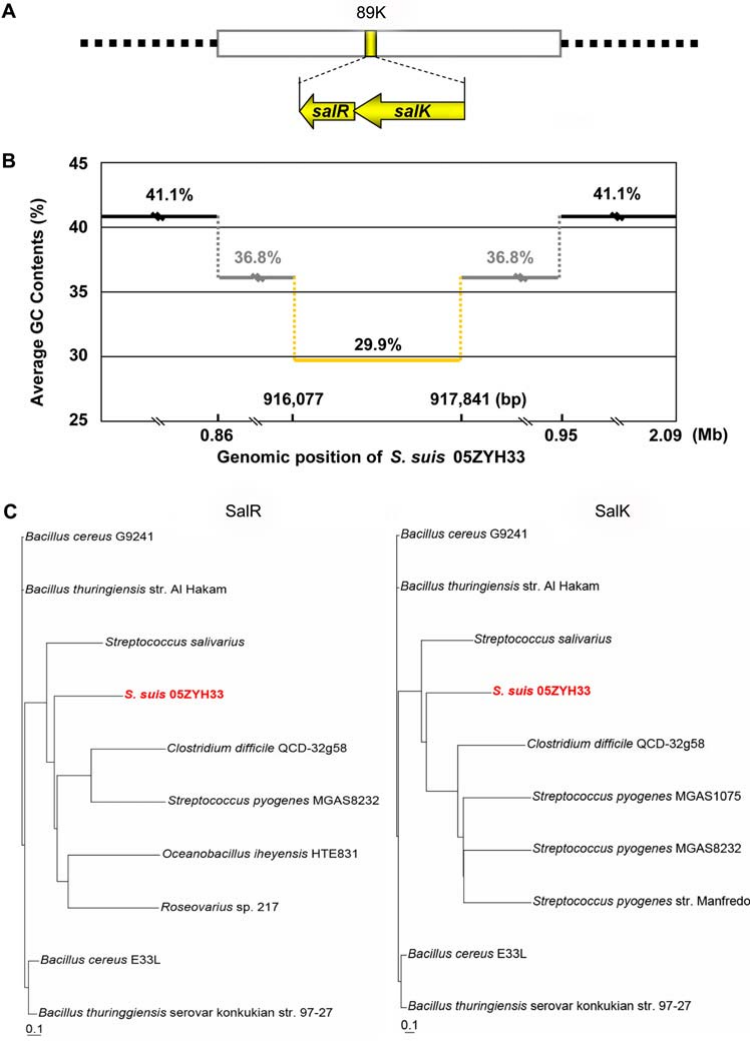
Identification and characterization of a unique two-component regulatory system SalK/SalR in the putative 89K PAI. A), Discovery of a unique two-component signal transduction system (TCSTS), SalK/SalR from 89K island. B), Aberrant average GC content of *salKR* which is much less than that of 89K and the whole genome. C), Phylogenetic analysis of the response regulator SalR and the sensor histidine kinase SalK of SalK/SalR regulatory system, with some related members known at the level of amino acids.

The newly decoded genomic sequence of *S. suis* 05ZYH33 makes it possible to systematically investigate the genetic basis of streptococcal pathogenicity. We focused on the putative 89K PAI to perform further molecular analysis. On the negative strand of 89K, peptides encoded by *05SSU0944* and *05SSU0943* exhibit 27% and 41% amino acid sequence identity with the SalK and the SalR proteins of *Streptococcus salivarius*, respectively, forming a TCSTS belonging to the Nar/LuxR family. *05SSU0944* and *05SSU0943* were accordingly renamed *salK* and *salR* (Fig. 1). Notably, the average GC content of *salKR*, 29.9%, is much less than that of both 89K and the whole genome of 05ZYH33 (36.8% and 41.1%, respectively) (Fig. 1B). The results of bioinformatics analysis indicated that the response regulator, SalR, contains 199 amino acids (aa) with the three N-terminal invariant aa residues (D30, D54 and K107) of the receiver domain of response regulators from Gram-positive bacteria [25]. In the C-terminal domain, it contains two residues (E142, G152) that are absolutely conserved in the winged helix-turn-helix DNA-binding domains of response regulators belonging to the LuxR family [26]. *salK* encodes a 396 aa long protein, with five N-terminal transmembrane domains and a transmitter domain signature motif in the C-terminal - typical of sensor histidine protein kinases of TCSTSs [25].

**Figure 2:**
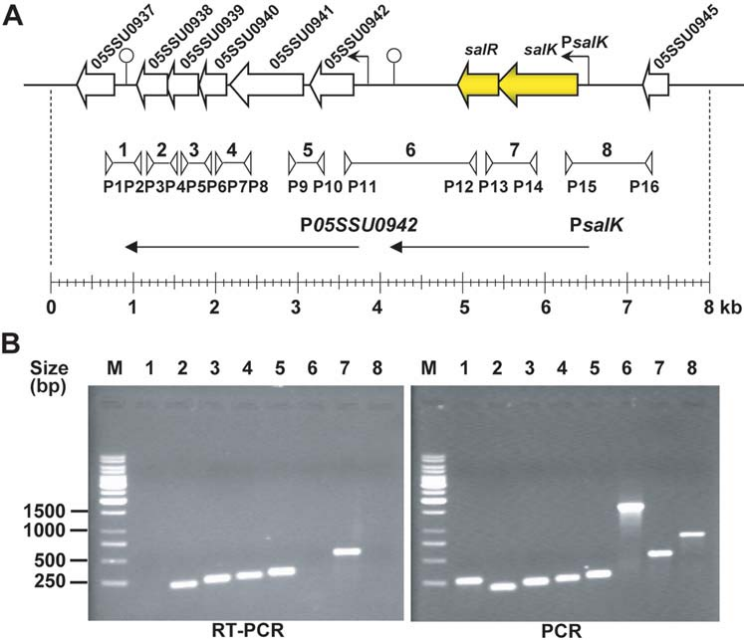
Transcriptional analysis of the *salKR* locus and its flanking genes of *S. suis* 05ZYH33. A), Genomic organization of the *salKR* locus and its flanking genes in *S. suis* 05ZYH33. Arrows represent the length and direction of transcription of the genes surrounding *salKR*. The putative transcripts initiated at P*05SSU0942* and P*salK* are depicted by thin horizontal arrows. Stem-loop structures represent relevant potential transcriptional terminators. The location of the primer pairs used in RT-PCR analysis are indicated by inverted arrowheads. B), RT-PCR analysis of the *salKR* locus and its flanking genes of 05ZYH33. Total RNA extracted from mid-exponential-phase cultures, and genomic DNA were analysed by RT-PCR and PCR, respectively, using primer pairs P1/P2 (lane 1), P3/P4 (lane 2), P5/P6 (lane 3), P7/P8 (lane 4), P9/P10 (lane 5), P11/P12 (lane 6), P13/P14 (lane 7) and P15/P16 (lane 8). The 1 kb DNA ladder marker is shown to the left (M).

As some TCSTS gene pairs are adjacent to the genes that they control, the genetic structure of the *salKR* locus and its flanking genes was defined (Fig. 2A). The *salK* coding region overlaps that of *salR* by 20 base pairs (bp), and no putative promoter or transcriptional terminator could be identified within either gene. A putative promoter located approximately 50 bp upstream from the TTG initiation codon of *salK*, was predicted using the online server of Neutral Network Promoter Prediction. Further sequence analysis revealed a palindrome of 55 bp with the potential to form a stem-loop structure situated in the intergenic region between *salR* and *05SSU0942*, which may function as a transcriptional terminator. *05SSU0945*, situated upstream (729 bp long intergenic region) of the *salKR* locus, encodes a 94 aa polypeptide for which there were no significant database matches. Downstream of *salKR*, the *05SSU0942*, *05SSU0941*, *05SSU0940*, *05SSU0939*, *05SSU0938* genes were predicted to encode peptides with significant identities to the orthologues of *Streptococcus agalactiae*. The function of these downstream gene products are unknown except for *05SSU0941*, which encodes a predicted DNA primase that might be involed in DNA replication. The genetic organization of this locus indicates that *salK* might be cotranscribed with *salR* initiating from the P*salK* promoter.

### Transcriptional Analysis of the *salKR* Locus and its Flanking Sequences

To confirm the predicted transcripts of *salKR* and the flanking genes, RNA extracted from *S. suis* 05ZYH33 cells was subjected to RT-PCR analysis by using primers amplifying each intergenic region of *salKR* and the flanking genes. As shown in Fig. 2B, RT-PCR products were obtained with five pairs of the primers (P3/P4, P5/P6, P7/P8, P9/P10 and P13/P14), but not with the other three primer pairs (P1/P2, P11/P12 and P15/P16). On the other hand, when genomic DNA was used as a template, all these primer pairs yielded the expected PCR products (Fig. 2B, PCR). These results suggested that *salK* and *salR* are transcriptionally coupled, and the five downstream (*05SSU0942-05SSU0938*) genes are co-transcribed from a promoter positioned immediately upstream of *05SSU0942* and that their transcription ends between the *05SSU0938-05SSU0937* intergenic region which contains a terminator-like inverted repeat sequence.

### Construction of Δ*salKR*

**Figure 3:**
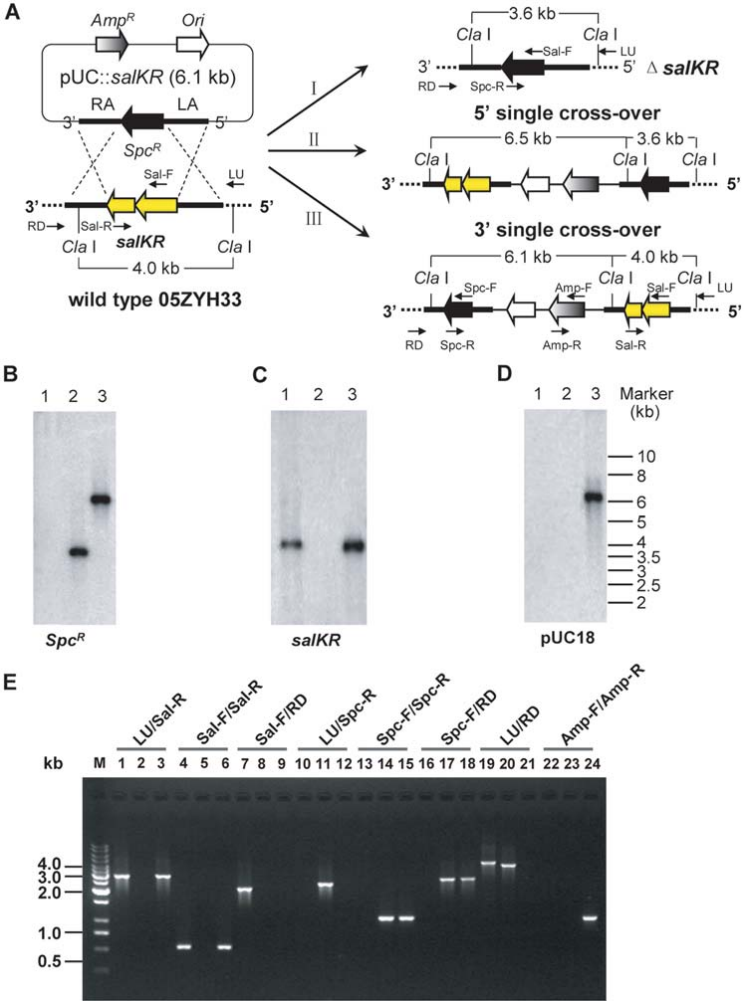
Construction and confirmation analysis of the knockout mutant strain Δ*salKR.* A), Strategy for deletion mutagenesis of *salKR* in *S. suis* 05ZYH33 by allelic replacement with a spectinomycin resistance cassette and schematic representation of the chromosomal structures before (left) and after (right) double cross-over (I) and single cross-over (II and III) recombination events between pUC::*salKR* and the chromosome of *S. suis* 05ZYH33. The location of the primers used in multiple-PCR detection are indicated by inverted arrowheads. The dotted lines represent the chromosomal sequences flanking the left and right arms of the construct. B, C and D), Southern hybridization analysis of the *salKR* region of *S. suis* wild type strain 05ZYH33 (lane 1), Δ*salKR* mutant (lane 2), and a 3′ single cross-over mutant with pUC::*salKR* integrated into the chromosome of 05ZYH33 (lane 3). Genomic DNA from each strain was digested with *Cla* I and separated on 0.7% agarose gel. The hybridization probes used were as follows: *Spc^R^* gene (B), an internal fragment of *salKR* (C), and pUC18 (D). E), Multiple-PCR analysis of the Δ*salKR* mutant. The primer combinations used in PCR are presented upon the lanes. Genomic DNA from the following strains were used as templates: wild type strain 05ZYH33 (lane 1, 4, 7, 10, 13, 16, 19 and 22), Δ*salKR* mutant (lane 2, 5, 8, 11, 14, 17, 20 and 23) and the 3′ single cross-over mutant (lane 3, 6, 9, 12, 15, 18, 21 and 24). The 1 kb DNA ladder marker is shown to the left (M). Theoretical size (bp) of each of the PCR products generated with the primer combinations was shown in Table S2.

To test the role of SalK/SalR in the pathogenesis of SS2, we constructed a homologous suicide plasmid, pUC::*salKR* with a *Spc^R^* cassette (Fig. 3A), and electrotransformed the competent cells of 05ZYH33. Positive transformants were first screened on THB agar plates under the selective pressure of spectinomycin. Colony PCR-based assays were performed to detect whether *salKR* was still present in the genome or not. One candidate mutant in which *salKR* failed to be amplified was obtained. To check this suspected mutant, Southern hybridization analyses were employed using probes of *Spc^R^* gene, an internal fragment of *salKR*, and pUC18, respectively. In wild type strain 05ZYH33, the internal fragment of *salKR* probe hybridized with a single 4.0 kb *Cla* I fragment (Fig. 3C, lane 1), whereas the *Spc^R^* and pUC18 probes did not hybridized with the genomic DNA (Fig. 3B and 3D, lane 1). In the suspected mutant, a 3.6 kb restriction fragment hybridized with the *Spc^R^* probe (Fig. 3B, lane 2), while no hybridization signal was detected when the internal fragment of *salKR* and pUC18 served as the probes (Fig. 3C and 3D, lane 2). In a single cross-over mutant which was set as the reference, both the *Spc^R^* and pUC18 probe hybridized with a 6.1 kb *Cla* I restriction fragment (Fig. 3B and 3D, lane 3), and the *salKR* probe hybridized with a 4.0 kb fragment (Fig. 3C, lane 3), indicating that this strain had pUC::*salKR* integrated into its chromosome by a single cross-over event at the 3′-flanking region of the *salKR* genes (Fig. 3A, III). Additionally, the allelic replacement of the wild-type *salKR* genes by the *Spc^R^* cassette in the suspected mutant was also confirmed by multiple-PCR analysis and sequencing with primers specific to genomic regions lying out the homologous left and right arms (Fig. 3A and 3E). All these results demonstrate that an isogenic knockout mutant of *salKR* (namely Δ*salKR*) was successfully constructed. RT-PCR experiments later also showed that neither *salK* nor *salR* could be transcribed in Δ*salKR* (data not shown), which further confirmed that *salKR* had been deleted from the bacterial chromosome.

### Role of SalK/SalR in Virulence

**Figure 4:**
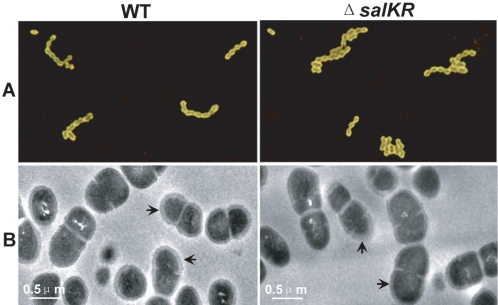
Cell morphology and ultrastructure of *S. suis* wild type strain 05ZYH33 and mutant strain Δ*salKR.* A), Morphology of bacteria under the light microscope using India ink staining (×1000). B), Transmission electron micrographs of bacteria. The capsule is highlighted by black arrows. The bar indicates the magnification size.

Given the multiple roles of TCSTS in bacteria, we evaluated the effect of *salKR* deletion on the general biological characteristics of SS2 prior to in vivo work. First, the ability of the mutant strain to retain spectinomycin resistance was assessed. The spectinomycin resistance phenotype was stable in Δ*salKR* during in vitro culture (data not shown). Next, the growth kinetics curves of Δ*salKR* were compared with that of WT, and no obvious distinctions were observed. When streaked on THB plates with 5% sheep blood, Δ*salKR* and WT colonies displayed a similar haemolytic phenotype. Finally, cell morphology and ultrastructure of these two strains were examined under light microscope and transmission electron microscope respectively, however, no significant differences were found (Fig. 4).

**Figure 5:**
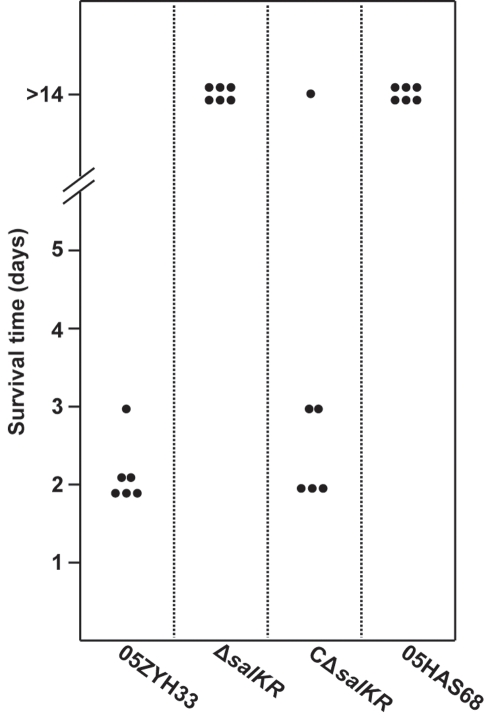
SPF-piglet experimental infections. Groups of 6 piglets were challenged intravenously with *S. suis* wild type strain 05ZYH33, mutant strain Δ*salKR*, complemented strain CΔ*salKR*, and avirulent strain 05HAS68, respectively, at a dose of 10^8^ CFU/piglet. Survival time (days) of individual piglets is indicated.

An experimental infection model in piglets was designed to assess the role of SalK/SalR in virulence. As shown in Figure 5, all six SPF-piglets inoculated intravenously with WT developed most of the typical disease symptoms, including high fever, limping, swollen joints, shivering, CNS failure and respiratory failure within 24 hrs. Five of them died on day 2 post-infection, and the last one on day 3. In contrast, six infected piglets with an avirulent strain, 05HAS68 (as negative control), survived nearly without any obvious symptoms until the end of the experiment (14 days after infection). Wherein the six piglets challenged with the Δ*salKR* mutant were all alive and symptom-free during the entire course monitored. This was similar to those observed in the negative control group, indicating that Δ*salKR* totally loses its lethality. The experiments of functional complementation with the complemented strain CΔ*salKR* showed clearly that the reintroduction of *salKR* into Δ*salKR* restored its high pathogenicity. Five out of six piglets succumbed to the lethal infections of CΔ*salKR* and died, with median survival times of 2 to 3 days. The last one recovered from serious clinical symptoms and survived at the experiments end. All these results strongly suggest that SalK/SalR plays an important role in the pathogenesis of Chinese isolates of highly invasive SS2.

To better evaluate the virulence attenuation of Δ*salKR*, colonization experiments were carried out. As shown in Table 1, wild-type bacteria were found in all of the SS2 specific tissues of WT-infected piglets, including heart, lung, liver, kidney, spleen, tonsil, brain, blood, joints and so on. In contrast, in the mutant-inoculated group, no mutant bacteria could be recovered from any of the examined tissue specimens. Obviously, lack of SalK/SalR badly impaired the capability of 05ZYH33 to colonize the various tissues of piglets. In the WT/Δ*salKR* coinfection group, besides the detection of wild-type bacteria in all the specimens, Δ*salKR* mutant bacteria could also be isolated from some susceptible tissues (such as heart, kidney, tonsil and brain) at a lower degree than the wild-type strain. We anticipated that the Δ*salKR* mutant is still able to colonize these organs, is just due to the WT-mediated impairment of host defense against SS2 infection in these tissues.

**Table 1:** Colonization analysis of Δ*salKR* in various tissues of piglets (CFU/g tissue).

Tissue	WT infection Group			WT/Δ*salKR* coinfectionGroup						Δ*salKR* infection Group		
	1#	2#	3#	4#		5#		6#		7#	8#	9#
	WT	WT	WT	WT	Δ*salKR*	WT	Δ*salKR*	WT	Δ*salKR*	Δ*salKR*	Δ*salKR*	Δ*salKR*
tonsil	3.4×10^6^	6.7×10^5^	8.6×10^5^	3.2×10^5^	1.8×10^5^	6.1×10^5^	4.3×10^4^	1.5×10^6^	9.7×10^4^	(-)	(-)	(-)
joint	4.9×10^5^	1.5×10^5^	7.4×10^5^	2.1×10^5^	(-)	8.6×10^4^	(-)	3.4×10^5^	(-)	(-)	(-)	(-)
CSF	6.3×10^4^	4.8×10^4^	1.1×10^5^	5.3×10^4^	(-)	1.3×10^4^	(-)	8.6×10^3^	(-)	(-)	(-)	(-)
heart	3.1×10^4^	8.2×10^4^	5.8×10^4^	1.1×10^4^	2.4×10^3^	2.8×10^4^	1.5×10^4^	6.6×10^3^	5.1×10^3^	(-)	(-)	(-)
liver	2.4×10^5^	5.4×10^4^	3.7×10^4^	7.5×10^3^	(-)	3.2×10^4^	(-)	6.4×10^4^	(-)	(-)	(-)	(-)
spleen	7.6×10^3^	2.3×10^4^	4.2×10^3^	3.2×10^3^	(-)	1.1×10^3^	(-)	5.1×10^3^	(-)	(-)	(-)	(-)
lung	1.9×10^4^	3.3×10^4^	5.0×10^4^	7.3×10^3^	(-)	3.6×10^4^	(-)	4.9×10^4^	(-)	(-)	(-)	(-)
kidney	8.5×10^4^	6.9×10^4^	1.7×10^5^	1.6×10^4^	4.3×10^3^	2.3×10^4^	2.1×10^4^	7.8×10^4^	3.7×10^4^	(-)	(-)	(-)
blood	7.2×10^5^	1.6×10^6^	6.6×10^5^	3.6×10^5^	(-)	4.1×10^5^	(-)	6.7×10^5^	(-)	(-)	(-)	(-)
brain	3.7×10^4^	2.5×10^4^	8.3×10^4^	4.1×10^4^	6.7×10^3^	7.3×10^3^	3.6×10^3^	3.2×10^4^	2.0×10^3^	(-)	(-)	(-)

### Decreased Resistance of Δ*salKR* to PMN-Mediated Killing

**Figure 6:**
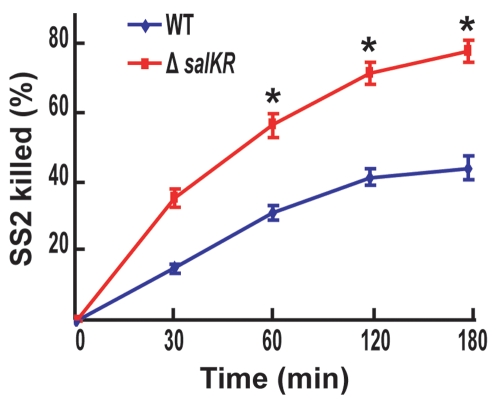
Decreased resistance of Δ*salKR* to PMN-mediated killing. SS2 wild type strain 05ZYH33 and mutant strain Δ*salKR* were co-incubated respectively with pig neutrophils (PMNs) at a moi of 10∶1. At each time, PMNs were lysed and bacteria were plated on growth agar. Colonies were enumerated the next day, and percent bacteria killed was calculated. Data are expressed as the mean±SD of three independent experiments. *, *P*<0.05.

Together with the wild type strain, Δ*salKR* was subjected to PMN-mediated killing assays. We found that the mortality rate of both strains (WT and Δ*salKR*) increased coordinately with the extension of the co-culture time with PMN cells (Fig. 6). However, Δ*salKR* had a higher mortality rate than that of WT, which means that Δ*salKR* was less able to resist killing by PMNs, key mediators of innate immunity. This result may account partially, if not most of all, for the virulence loss in Δ*salKR*.

### Expression Microarray Analysis of the Mutant Strain Δ*salKR*

To gain further insights into the network/circuit mediated by SalK/SalR, whole-genome DNA microarray was applied to reveal the differential transcription profiles between Δ*salKR* and WT [13], [19]. For identifying genes whose expression was significantly different, the threshold for the expression ratio between the mutant and wild type strain was set at 2. In total, the absence of SalK/SalR led to decreased expression of 26 genes spread throughout the genome (i.e. 1.2% of the 2194 tested). These down-regulated genes can be roughly categorized into the 3 groups, which can be found in Table 2.

**Table 2:** Collection of the down-regulated genes in Δ*salKR*.

Gene	Δ*salKR*/WT	Annotation	Function category	Predicted transcript
05SSU1547	0.161	Amino acid ABC-type transport system, permease	Amino acid transport and metabolism	Cotranscribes with 05SSU1546–05SSU1543
05SSU1725	0.257	Permeases of the major facilitator superfamily	Carbohydrate transport and metabolism	Monocistron
05SSU0451	0.389	Phosphotransferase system	Carbohydrate transport and metabolism	Monocistron
05SSU0282	0.414	ABC transporter ATP-binding protein	Carbohydrate transport and metabolism	Cotranscribes with 05SSU0283–05SSU0286
05SSU1489	0.486	6-phospho-beta-glucosidase	Carbohydrate transport and metabolism	Monocistron
05SSU1394	0.496	Permeases of the major facilitator superfamily	Carbohydrate transport and metabolism	Cotranscribes with 05SSU1393 and 05SSU1392
05SSU1731	0.435	Probable permease	Carbohydrate transport and metabolism	Cotranscribes with 05SSU1730 and 05SSU1729
05SSU1103	0.395	Phosphate ABC transporter	Inorganic ion transport and metabolism	Cotranscribes with 05SSU1106–05SSU1100
05SSU1009	0.400	Orotidine-5-phosphate decarboxylase	Nucleotide transport and metabolism	Cotranscribes with 05SSU1008–05SSU1003
05SSU1440	0.480	Acyl-ACP thioesterase	Lipid transport and metabolism	Cotranscribes with 05SSU1439 and 05SSU1438
05SSU0293	0.474	ABC-type multidrug transport system, ATPase	Defense system	Monocistron
05SSU0090	0.485	Preprotein translocase subunit SecY	Intracellular trafficking and vesicular transport	Monocistron
05SSU0063	0.275	Recombination protein A	Recombination and repair	Cotranscribes with 05SSU0064
05SSU0953	0.493	DNA recombinase	Recombination and repair	Monocistron
05SSU0588	0.494	Transposase and inactivated derivatives	Recombination and repair	Monocistron
05SSU0503	0.227	Mercuric resistant regulatory protein	Transcription	Monocistron
05SSU1233	0.498	Probable surface antigen negative regulator Par	Transcription	Monocistron
05SSU0935	0.330	Hypothetical protein	Unknown	Cotranscribes with 05SSU0934
05SSU1912	0.347	Predicted membrane protein	Unknown	Monocistron
05SSU0677	0.444	Predicted membrane protein	Unknown	Monocistron
05SSU2088	0.354	Hypothetical protein	Unknown	Monocistron
05SSU0775	0.366	Hypothetical protein	Unknown	Monocistron
05SSU1126	0.423	Uncharacterized conserved protein	Unknown	Monocistron
05SSU1831	0.467	Hypothetical protein	Unknown	Monocistron
05SSU0374	0.492	Homolog of plant Iojap protein	Unknown	Monocistron
05SSU0108	0.402	Hypothetical protein	Unknown	Monocistron

#### Genes involved in substance transport and metabolism

In the Δ*salKR* mutant, transcripts of several ABC-transporters are less abundant. As learned, ABC-transporters and permeases are usually involved in controlling influx and efflux of small molecules through metabolic pathways in response to environmental changes in bacteria [27]. Multidrug resistance [28], [29] and pathogenicity [10] can also be attributed to ABC-transporters. It is widely accepted that over-expression of ABC-type multidrug transporters can efficiently evade the pressure of drugs and enhance the invasion ability of pathogens [28]–[30]. Furthermore, orotidine-5-phosphate decarboxylase (05SSU1009), a newly identified pathogenesis-related protein in *Vibrio vulnificus* [31], was also found to be repressed greatly in Δ*salKR*. Daigle *et al.* [32] has shown that mutation of phosphotransferase system (PST) in extraintestinal pathogenic *E. coli* (ExPEC) can cause the loss of its colonization ability in extraintestinal organs, and bacteria are cleared rapidly from the bloodstream. Similarly, we observed that *pst* (*05SSU0451*) is down-regulated in Δ*salKR* whose survival in the bloodstream is drastically affected (Fig. 6).

#### Genes revolved in recombination/repair and transcription

Together with some elements of DNA recombination/repair (05SSU0063, 05SSU0588 and 05SSU0953), two transcription regulators (05SSU0503 and 05SSU1233) were also under the regulation of SalK/SalR. Although these genes were found to be down-regulated in the mutant, it is not understood how they are associated with the attenuation of Δ*salKR*.

#### Genes encoding proteins of unknown function

Several membrane proteins such as 05SSU0677 and 05SSU1912 were also repressed in Δ*salKR*. It is well documented that surface proteins or cell wall-anchored proteins are usually thought to contribute to the immunogenicity [33], [34], invasiveness [35]–[37], and even pathogenesis [38], [39] of pathogens. Moreover, it should be noted that numerous proteins of unknown function are also depressed in Δ*salKR*, which may in part lead to the virulence loss of the mutant via some thus-far-unknown mechanisms.

Remarkably, no gene was found to be up-regulated (more than twice) in Δ*salKR*. To examine the validity of the microarray data, we performed further quantitative real-time PCR analysis on 42 genes using RNA from three independent cultures of each strain (WT, the Δ*salKR mutant* and the complemented strain CΔ*salKR*). These genes include the total 26 ones that are down-regulated in the Δ*salKR* mutant, four virulence factors (CPS, MRP, EF, SLY) coding genes [40]–[44], genes immediately flanking the *salKR* locus (*05SSU0945* and *05SSU0942*), and 10 genes that selected from the chromosome of 05ZYH33 every 200 ORFs. As shown in Table S1, the quantitative results of RT-PCR were highly compatible to the microarray data, in the Δ*salKR* mutant, 41 out of 42 selected genes (97.6%) were confirmed to have similar transcript levels using these two different techniques, thus independently confirming the microarray results. The discrepancies of target genes expression between microarray data and real-time quantitative PCR results probably best reflects differences in the relative sensitivity and specificity of the two methods. Among the 26 down-regulated genes in the Δ*salKR* mutant, five did not recover to expression level of the wild-type strain, indicating that the complemented strain does not imitate completely the exact tuning of gene expression as in the wild-type strain.

## Discussion

Since the first description of human infection in Denmark in 1968, SS2 seems to have developed some unknown machinery to behave much more invasively, which is supported by two recent large-scale outbreaks of human epidemics in China (one had 25 cases with 14 deaths in Jiangsu in 1998, the second had 204 cases with 38 deaths in Sichuan in 2005) [4], [5]. During the past decades, a series of virulence determinants of SS2 involved in the survival, spread and pathopoiesis of the bacterium within the host have been identified, such as capsule polysaccharide (CPS), muramidase-related protein (MRP), suilysin, etc. Very recently, Chen *et al.* [6] reported a candidate PAI of ∼89 kb in the epidemic outbreak strains of SS2 infections in China, gave some genomic clues for elucidating molecular pathways by which SS2 behaved so aggressively. However, they did not provide sufficient experimental evidence supporting the prediction of 89K PAI which was largely based on the results of bioinformatical analysis, and the functions of the vast majority of genes encoding within the 89K island remain largely unknown.

In this study, we carried out a detailed structural and functional analysis of a unique two-component regulatory system designated SalK/SalR which is located in the putative 89K PAI. Bioinformatics analysis revealed that the GC content of *salKR* is far less than that of 89K island and the whole genome, implying that this TCSTS is of foreign origin which may have been acquired through horizontal gene transfer. It has been well documented that horizontal gene transfer events occur frequently in pathogens [45]–[47], and some of them have been implicated in high pathogenicity [48] and even in the causation of severe diseases [49]. Suilysin, a known virulence factor, was believed to be introduced into SS2 via lateral gene transfer [43]. Moreover, PCR analysis using primers amplifying the *salKR* locus also demonstrated that this TCSTS is specific to Chinese epidemic outbreak strains of SS2 (data not shown), implying its tight linkage to the virulence of this new emerging pathogenic species.

In order to define the role of SalK/SalR in SS2 infection, an isogenic SalK/SalR-deficient mutant was generated and the impact of *salKR* deletion on virulence of SS2 was assessed. Results of piglets experimental infections clearly showed that the deletion of *salKR* leads to elimination of the lethality of this important pathogen. Colonization analysis also revealed the incapability of the Δ*salKR* mutant to colonize any susceptible tissue of piglets when administered alone. These results indicate definitely that SalK/SalR plays a critical role in the pathogenicity of SS2. The reduction in virulence was mostly restored in a complemented strain upon reintroduction of functional copies of *salK* and *salR* into the mutant, suggesting that it did not result from polar effects on expression of flanking genes. We consider that the partial complementation observed in the piglets infection model is inherent to the pleiotropic role of this regulatory system which makes it difficult to restore the fine tuning of gene expression as in the wild-type strain. Results of real-time quantitative RT-PCR also confirmed that, in the complemented strain CΔ*salKR*, only partial genes identified as down-regulated in the mutant rebounded to comparative transcript levels of the wild-type strain. Those unrecovered genes were probably irrelavent to the bacterial virulence of SS2.

It is known that TCSTSs are often implicated in the regulation of the direct downstream genes. However, genes immediately flanking the *salKR* locus were identified as transcriptionally unaffected by the disruption of the *salKR* genes. This result is in agreement with the predicted operon organization in the genome, which suggests that *salK* and *salR* are a single co-transcriptional unit. Among the 26 target genes affected by SalK/SalR, 17 of them (65.4%) were predicted to be monocistronically transcribed, the others to be part of larger transcriptional units (Table 2). For those members belonging to putative transcriptional operons, microarray results showed that most of those genes cotranscribed with them were also down-regulated to a various degree, although under the threshold of 2. In the 89K island, transcripts of only two genes designated *05SSU0935* and *05SSU0953* that encode a hypothetical protein and a DNA recombinase respectively, were decreased in Δ*salKR*, their contributions to the attenuation are unknown. It seems that SalK/SalR may control the full virulence of SS2 by regulating distant gene expression at the genome-wide level. Further investigation of other transcriptional alterations observed in the microarray data will be required for a better understanding of pathogenesis of this highly pathogenic pathogen. Efforts are currently being made in order to address functions of those down-regulated hypothetical proteins individually.

It has been described that genes essential for viability and important virulence factors are often under the regulation of TCSTS. However, in our study, neither microarray findings nor further analysis of real-time quantitative RT-PCR revealed any differences in level of transcription of the known virulence factors (CPS, suilysin, MRP and EF) between the wild type and mutant strain. Several possible explanations may be envisaged. First, some pathogenic determinants are environmentally regulated and induced at specific stages of the infection process [50], [51]. Secondly, some of the TCSTSs of *S. pneumoniae* were suggested to be involved in key processes of pathogenesis, such as autolysis and cell-cell signaling [52], SalK/SalR might regulate similar processes in Chinese SS2 virulent isolates. More importantly, accumulated evidence suggests that some of the known virulent factors are not requisite for the full virulence of SS2, as the absence of one or more of these proteins in isolates from infected animals cannot necessarily be associated with a lack of virulence [53]–[56].

In conclusion, a unique two-component regulatory system SalK/SalR has been identified from a putative 89K PAI in Chinese isolates of highly pathogenic SS2. Prior to this study, SalK/SalR is only known to be associated with the production of a kind of lantibiotic peptide named salivaricin A (SalA) in *S. salivarius* [11], as yet, this TCSTS has not been linked to the regulation of bacterial virulence. Our data confirm, for the first time, that SalK/SalR is absolutely indispensable for the full virulence of Chinese highly invasive SS2 strains, although we fails to define its precise regulatory mechanism. Not only does this investigation provide experimental evidence for the validity of the candidate 89K PAI, but it adds novel insights into the infectious disease pathogenesis of SS2.

## Materials and Methods

### Bacterial Strains, Plasmids and Growth Conditions

Bacterial strains and plasmids used in this study are listed in Table 3. SS2 strains were grown in Todd-Hewitt broth (THB) (Difco Laboratories, Detroit, MI) medium or plated on THB agar containing 5% (vol/vol) sheep blood. *E. coli* DH5α was cultured in Luria Broth (LB) liquid medium or plated on LB agar. When necessary, 100 µg/ml of spectinomycin (Spc) (Sigma) or 5 µg/ml of chloromycetin (Cm) (Sigma) was used for *S. suis* transformants, and 50 µg/ml of ampicillin (Amp) (Sigma) was applied to screen the transformants of *E. coli*.

**Table 3:** Summary of bacterial strains and plasmids.

Strains/plasmids	Characteristics/function*	Source/reference
**Bacterial strains**		
05ZYH33	Virulent strain of SS2 isolated from dead patients with a toxic shock-like syndrome	Collected in our laboratory
Δ*salKR*	The deletion mutant of *salKR* with background of 05ZYH33, Spc^R^	In this study
CΔ*salKR*	Complemented strain of Δ*salKR*; Spc^R^; Cm^R^	In this study
05HAS68	Avirulent SS2 isolated from a healthy pig	Collected in our laboratory
*E. coli* DH5α	Cloning host for maintaining the recombinant plasmids	Promega
**Plasmids**		
pUC18	Cloning vector; Amp^R^	Promega
pSET1	*E. coli-S. suis* shuttle vector; Cm^R^	[65]
pSET2	*E. coli-S. suis* shuttle vector; Spc^R^	[65]
pUC::*salKR*	A recombinant vector with the background of pUC18, designed for knockout of *salKR*; Amp^R^ ; Spc^R^	In this study
pSET::C	pSET1 inserted with the intact *salKR* genes and the upstream promoter; Cm^R^; Spc^R^	In this study

* Amp^R^, ampicillin resistant; Cm^R^, chloromycetin resistant; Spc^R^, spectinomycin resistant.

### Construction of *S. suis* Mutant, Δ*salKR*

To test the role of SalK/SalR in the pathogenesis of SS2, *salKR* was activated by allelic replacement with a constitutively expressed spectinomycin resistance (*Spc^R^*) cassette. First, the flanking DNA sequences to *salKR* were amplified from the chromosomal DNA of *S. suis* 05ZYH33 using PCR technique with two pairs of specific primers (LA-F1/LA-R1 and RA-F1/RA-R1) carrying *Eco*R I/*Bam*H I and *Pst* I/*Hin*d III restriction enzyme sites, respectively (Table 4). Followed by digestion with the corresponding restriction enzymes, the DNA fragments were cloned into a pUC18 vector directionally. Then, the *Spc^R^* gene cassette (from pSET2) was inserted at the *Bam*H I/*Pst* I sites to generate the *salKR* knockout vector pUC::*salKR*. To obtain the isogenic mutant Δ*salKR*, the competent cells of 05ZYH33 were subjected to electrotransformation with pUC::*salKR* as described by Smith *et al.* [57]. For all the *Spc^R^* transformants, colony PCR assay was used to examine them with a series of specific primers. To further confirm whether the suspected mutant was caused by double cross-over recombination, Southern blotting analysis was carried out. The DNA probes including *Spc^R^* gene, an internal fragment of *salKR*, and pUC18 were labeled with biotin using the random primed DNA labeling kit (Pierce). The hybridization signals were detected with the hybridization and detection kit (Pierce) according to the standard protocol recommended by the manufacturer.

**Table 4:** Primers used for PCR amplification and detection.

Primers	Sequence of primers (5′-3′)	Restriction sites	Functions
P1	ACAAATACGGTCGATGAGTTC	/	For transcription analysis
P2	TTGGAGAAGCGAATAGAGCAG	/	
P3	AAAGGTAGCCCGCATAGGAC	/	For transcription analysis
P4	ACAAAGTCAGCCGAAGAAGGTA	/	
P5	GTTCAAAAGCAGCCACATAGTC	/	For transcription analysis
P6	TATCAAACGGCAACAATCAAGT	/	
P7	TTCTTGCCTAGCTACTTTTTCA	/	For transcription analysis
P8	TCGTTCAAGATTTACCACCAT	/	
P9	AACCCAAACTTTCAGCCACATC	/	For transcription analysis
P10	ACAACAGCGAACCACTCAGACA	/	
P11	TTAAACCTCCTACTCCAATCAG	/	For transcription analysis
P12	AAATGGGGAAAAACGAAAAG	/	
P13	TTTCTTTTTCGCGTTCTGTC	/	For transcription analysis
P14	GGGGGACTATTACTTTTGAG	/	
P15	TCACAAATAGATAAAAAGAAGT	/	For transcription analysis
P16	TAAAAGGAATATCTAGTCAACAG	/	
LA-F1	CT**GAATTC**ATGTGTTCCCGATAA ATGGTA	*Eco*R I	Upstream border of *salKR*
LA-R1	GCA**GGATC**CTCACAAATAGATAAAAAGAAG	*Bam*H I	
RA-F1	GG**CTGCAG**CAGAACGCGAAAAAGAAATAC	*Pst* I	Downstream border of *salKR*
RA-R1	TGG**AAGCTT**CCATAAGACCTCCCTAATCAT	*Hin*d III	
Spc-F	GCA**GGATCC**GTTCGTGAATACATGTTATA	*Bam*H I	*Spc^R^* gene cassette
Spc-R	GG**CTGCAG**GTTTTCTAAAATCTGAT	*Pst* I	
Sal-F	GGGGGACTATTACTTTTGAG	/	an internal fragment of *salKR*
Sal-R	TTTCTTTTTCGCGTTCTGTC	/	
LU	AATATTTTTCAGCTTACTCACGA	/	For PCR assay
RD	AACTGACTGCGATTAAACCTCCTAC	/	
Amp-F	ATGTGCGCGGAACCCCTATTTG	/	For PCR assay
Amp-R	TCTTTTCTACGGGGTCTGACGC	/	
C-F	GCA**GGATCC**ATTGTTAACCCCTACTCCTATT	*Bam*H I	A fragment with *salKR*
C-R	CT**GAATTC**AACAAAACATCGACAACATACA	*Eco*R I	

The bold sequences are the restriction sites.

### Functional Complementation of Δ*salKR*

For complementation analysis, the following strategy was utilized. A DNA fragment containing *salKR* and its upstream promoter was amplified by PCR. Digested with the appropriate restriction enzymes, the resulting DNA fragment was cloned into the *E. coli-S. suis* shuttle vector pSET1 to generate the recombinant plasmid pSET::C. Then pSET::C was electrotransformed into the Δ*salKR* mutant to screen the complemented strain of Δ*salKR* (namely CΔ*salKR*) on THB agar with double selection pressure of Spc and Cm.

### Experimental Infections of Piglets

To evaluate the effect of *salKR* deletion on the virulence of 05ZYH33, three-week-old SPF-piglets (6 piglets/group) were challenged with Δ*salKR* and its complementary strain CΔ*salKR*. The virulent wild type (WT), 05ZYH33, and an avirulent strain, 05HAS68, were utilized as positive reference and negative reference, respectively [5]. As a matter of fact, 50% lethal doses should be ascertained in order to determine differences in virulence between the wild type and mutant strain. For ethical reasons, we could not do this kind of experiment in piglets. Instead, groups of six SPF-piglets were inoculated intravenously with the above 4 types of SS2 strains (dose of 10^8^ CFU/piglet) respectively. Clinical signs and survival time were then recorded. All animal experiments were performed in a facility of bio-safety level 3 (BSL-3) and approved by the local ethical committee.

### Colonization ability analysis of Δ*salKR* in susceptible tissues of piglets

To better define the difference between the WT strain and the Δ*salKR* mutant, the capability of each strain to colonize the various tissues of piglets was analyzed. Groups of three SPF-piglets were inoculated intravenously with the wild type strain 05ZYH33, Δ*salKR* mutant and a 1∶1 mixture of WT/Δ*salKR*, respectively, at a dose of 10^8^ CFU/piglet. When the WT-infected piglets developed typical SS2 infection symptoms, all these three groups of piglets were sacrificed for colonization analysis as previously described [58].

### PMN-mediated Killing Experiments

Polymorphonuclear leukocytes (PMNs, also called neutrophils) were isolated from heparinized venous blood of healthy piglets using the method of dextran sedimentation as described by Baltimore *et al.* [59]. After removing the contaminant erythrocytes by adding ice-chilled 0.2% NaCl, the excess neutrophils were resuspended in RPMI 1640 medium (Mediatech). The viabilities of neutrophils were confirmed using trypan blue dye exclusion. Killing experiments of SS2 by pig PMNs were carried out as described in earlier methods with minor modifications [60], [61]. Briefly, SS2 in exponential phase were washed, and resuspended in RPMI 1640. Bacteria were opsonized in 50% normal pig serum for 30 min at 37°C, and then concentrated to 10^8^ CFU in RPMI 1640. PMNs (10^6^) were combined with 10^7^ opsonized SS2 in 96-well plates on ice and centrifuged at 380× *g* for 5 min at 4°C. Samples were analyzed immediately. At each time, PMNs were lysed with 0.1% saponin (20 min on ice), and serial dilutions of the lysates were plated on THB agar. Colonies were counted and the percentage of SS2 killed was measured with the equation of [1−(CFU_PMN+_/CFU_PMN−_)]×100% [16]. Each assay was performed in triplicate.

### Microarray-based Comparative Transcriptomics Analysis of Δ*salKR*

According to the whole genome sequence of 05ZYH33, specific oligo-nucleotide probes with an average length of 35 nt were designed to cover every putative ORF (2194 in total) in SS2 genome using Array Designer 2.0 and then synthesized onto complementary metal oxide semiconductor (CMOS) matrix utilizing *in situ* electrochemical synthesis technique (Combimatrix). Total RNA was extracted from SS2 cultures grown in THB to OD_600_ of 0.8, with Trizol reagent (Invitrogen), and purified using RNeasy Mini Kit (Qiagen). Subsequently, cDNA was labeled with Cy3-dCTP during the reverse transcription. After pre-hybridization, microarray slides were hybridized with the relevant samples at 65°C for 12∼16 h [62]. This was repeated 4 times for both samples (WT and Δ*salKR*). All hybridization slides were scanned by GenePix 4100A scanner after appropriate washing, and the average pixel intensity values were quantified using GenePix Pro 4.1. Statistical analysis was performed, e.g., *t*-test and *P*-value. Finally, those genes with more than twice of change ratio were selected to be candidate targets [62].

### Real-time Quantitative RT-PCR Evaluation

To validate the data from microarray analysis, 42 genes were selected to confirm by real-time quantitative PCR with SYBR Green detection as described [63]. Quantitative analysis were performed in triplicate with SYBR premix Ex Taq™ (TaKaRa) on an Opticon 2 (MJ Research, USA) using RNA from three independent cultures of each strain (WT, Δ*salKR* and CΔ*salKR*). Glyceraldehyde-3-phosphate dehydrogenase (GAPDH) was utilized to serve as the internal control gene. Various *C*t values were normalized to average *C*t values for the endogenous reference transcript, GAPDH [63]. The mean fold changes in target gene expression were calculated as described by Livak *et al.* [64].

### Statistical Analysis

Where appropriate, the data were analyzed using Student's *t* test, and a value of *P*<0.05 was considered significant.

## Supporting Information

Table S1 Real-time quantitative RT-PCR validation of microarray data
(0.08 MB DOC)

Table S2 Theoretical size (bp) of each of the PCR products generated with the primer combinations used in the multiple-PCR analysis of ΔsalKR mutant
(0.03 MB DOC)
