# Supplementary material for: SalK/SalR, a Two-Component Signal Transduction System, Is Essential for Full Virulence of Highly Invasive Streptococcus suis Serotype 2
Source: PLoS One. 2008 May 7;3(5):e2080. doi: 10.1371/journal.pone.0002080 (PMC2358977; doi:10.1371/journal.pone.0002080)
Supplement: Table S1 — Real-time quantitative RT-PCR validation of microarray data (0.08 MB DOC) [file pone.0002080.s001.doc]

**SUPPORTING INFORMATION**

**Table S2.** Theoretical size (bp) of each of the PCR products generated with the primer combinations used in the multiple-PCR analysis of Δ*salKR* mutant

| templates | LU/Sal-R | Sal-F/Sal-R | Sal-F/RD | LU/Spc-R | Spc-F/Spc-R | Spc-F/RD | LU/RD | Amp-F/Amp-R |
| --- | --- | --- | --- | --- | --- | --- | --- | --- |
| 05ZYH33 | 2898 | 514 | 2142 | (-) | (-) | (-) | 4526 | (-) |
| Δ*salKR* | (-) | (-) | (-) | 2470 | 1130 | 2776 | 4116 | (-) |
| 3’ single cross-over mutant | 2898 | 514 | 8291^*^ | 9030^*^ | 1130 | 2776 | 10675^*^ | 1128 |

* too long to be obtained under conventional PCR conditions.
