# Supplementary material for: SalK/SalR, a Two-Component Signal Transduction System, Is Essential for Full Virulence of Highly Invasive Streptococcus suis Serotype 2
Source: PLoS One. 2008 May 7;3(5):e2080. doi: 10.1371/journal.pone.0002080 (PMC2358977; doi:10.1371/journal.pone.0002080)
Supplement: Table S2 — Theoretical size (bp) of each of the PCR products generated with the primer combinations used in the multiple-PCR analysis of ΔsalKR mutant (0.03 MB DOC) [file pone.0002080.s002.doc]

**SUPPORTING INFORMATION**

**Table S1. Real-time quantitative RT-PCR validation of microarray data**

| **Gene codes** | Annotation | **Change Ratio** | | |
| --- | --- | --- | --- | --- |
|  |  | Microarray(Δ*salKR*/WT) | Real time RT-PCR | |
|  |  |  | **(Δ*salKR*/WT)** | **(CΔ*salKR*/WT)** |
| 05SSU1547 | Amino acid ABC-type transport system, permease | 0.161 | 0.214 | 0.869 |
| 05SSU1725 | Permeases of the major facilitator superfamily | 0.257 | 0.282 | 0.943 |
| 05SSU0451 | Phosphotransferase system | 0.389 | 0.346 | 0.905 |
| 05SSU0282 | ABC transporter ATP-binding protein | 0.414 | 0.487 | 1.052 |
| 05SSU1489 | 6-phospho-beta-glucosidase | 0.486 | 0.323 | 0.743 |
| 05SSU1394 | Permeases of the major facilitator superfamily | 0.496 | 0.225 | 0.811 |
| 05SSU1103 | Phosphate ABC transporter | 0.395 | 0.302 | 0.781 |
| 05SSU1009 | Orotidine-5-phosphate decarboxylase | 0.400 | 0.453 | 1.134 |
| 05SSU1440 | Acyl-ACP thioesterase | 0.480 | 0.295 | 0.366 |
| 05SSU1731 | Probable permease | 0.435 | 0.374 | 0.412 |
| 05SSU0090 | Preprotein translocase subunit SecY | 0.485 | 0.352 | 0.793 |
| 05SSU0293 | ABC-type multidrug transport system, ATPase | 0.474 | 0.314 | 0. 968 |
| 05SSU0063 | Recombination protein A | 0.275 | 0.486 | 0.889 |
| 05SSU0953 | DNA recombinase | 0.493 | 0.363 | 1.187 |
| 05SSU0588 | Transposase and inactivated derivatives | 0.494 | 0.298 | 0.402 |
| 05SSU0503 | Mercuric resistant regulatory protein | 0.227 | 0.394 | 0.766 |
| 05SSU1233 | Probable surface antigen negative regulator Par | 0.498 | 0.421 | 0.920 |
| 05SSU0935 | Hypothetical protein | 0.330 | 0.456 | 1.025 |
| 05SSU1912 | Predicted integral membrane protein | 0.347 | 0.173 | 0.952 |
| 05SSU0677 | Predicted integral membrane protein | 0.444 | 0.372 | 0.816 |
| 05SSU2088 | Hypothetical protein | 0.354 | 0.601 | 0.768 |
| 05SSU0775 | Hypothetical protein | 0.366 | 0.492 | 0.947 |
| 05SSU1126 | Uncharacterized conserved protein | 0.423 | 0.471 | 0.483 |
| 05SSU1831 | Hypothetical protein | 0.467 | 0.288 | 1.454 |
| 05SSU0374 | Homolog of plant Iojap protein | 0.492 | 0.316 | 0.448 |
| 05SSU0108 | Hypothetical protein | 0.402 | 0.497 | 0.742 |
| 05SSU0945 | Hypothetical protein | 1.242 | 1.093 | 0.929 |
| 05SSU0942 | Hypothetical protein | 0.934 | 0.835 | 0.912 |
| 05SSU0573 | CPS2J | 1.311 | 1.124 | 0.766 |
| 05SSU0753 | Muramidase-released protein (MRP) | 1.014 | 0.835 | 0.917 |
| 05SSU0177 | Extracellular protein (EF) | 1.060 | 1.221 | 0.956 |
| 05SSU1403 | Suilysin (SLY) | 0.950 | 1.367 | 1.183 |
| 05SSU0200 | Pyruvate-formate lyase | 1.044 | 0.793 | 0.884 |
| 05SSU0400 | Hypothetical protein | 1.185 | 1.477 | 1.206 |
| 05SSU0600 | Putative prephenate dehydratase | 0.758 | 0.941 | 1.135 |
| 05SSU0800 | ABC-type multidrug transport system | 1.151 | 0.872 | 0.846 |
| 05SSU1000 | Putative 5'-nucleotidase | 0.984 | 0.728 | 1.042 |
| 05SSU1200 | Phosphoenolpyruvate-protein kinase | 1.066 | 1.245 | 1.312 |
| 05SSU1400 | Transcriptional regulators | 1.255 | 0.996 | 1.061 |
| 05SSU1600 | Osmoprotectant binding protein | 1.266 | 1.438 | 1.113 |
| 05SSU1800 | 3-hydroxymyristoyl/3-hydroxydecanoyl-(acyl carrier protein) dehydratases | 0.863 | 0.674 | 0.920 |
| 05SSU2000 | Permeases of the major facilitator superfamily | 0.782 | 0.736 | 0.852 |

* GAPDH, a housekeeping gene was selected to serve as the reference gene in the experiments of real-time quantitative RT-PCR. Data shown are the means of triplicate values and are representative of three independent experiments.
